# Insight Into the Role of NLRP3 Inflammasomes in Takayasu's Arteritis: Mechanisms and Targeted Pharmacotherapies

**DOI:** 10.31083/RCM38815

**Published:** 2025-10-28

**Authors:** Zhao-Yu Wang, Tian-Yi Ma, Kang Huang, Jiang-Hua Zhong, Shi-Juan Lu, Jian-Jun Li

**Affiliations:** ^1^Department of Cardiology, Haikou Affiliated Hospital of Central South University Xiangya School of Medicine, 570208 Haikou, Hainan, China; ^2^Cardiometabolic Center, State Key Laboratory of Cardiovascular Diseases, Fu Wai Hospital, National Center for Cardiovascular Diseases, Chinese Academy of Medical Sciences and Peking Union Medical College, 100037 Beijing, China

**Keywords:** Takayasu's arteritis, NLRP3 inflammasome, signaling pathway, treatment

## Abstract

Takayasu's arteritis (TAK) is a rare chronic arteritis that can lead to serious consequences. Understanding of the pathogenesis of TAK remains limited, and effective therapeutic strategies for this condition are lacking. Previous studies have suggested that there may be an association between TAK and the interaction between Mycobacterium tuberculosis infection and genetic susceptibility. Emerging data indicate that the nucleotide-binding and oligomerization domain (NOD)-like receptor pyrin domain-containing protein 3 receptor (NLRP3) inflammasome may be involved in the pathogenesis of TAK, potentially contributing to the initiation of the disease. This review summarizes the current epidemiological data, possible mechanisms, and targeting strategies of TAK, focusing on the involvement of the NLRP3 inflammasome in the pathogenesis of TAK, and provides new insights into the prevention and treatment of this condition.

## 1. Introduction

Takayasu’s arteritis (TAK) is a chronic inflammatory disease that primarily affects the aorta and its major branches, causing granulomatous inflammation and fibrosis of the vessel wall. It is a rare disease, but can lead to severe complications. TAK can affect large and medium arteries throughout the body, causing various non-specific complications such as visual impairment, stroke, myocardial infarction, heart failure, hypertension, and intermittent claudication. Laboratory tests, while indicating inflammation with elevated markers, are not specific to TAK, making diagnosis heavily reliant on angiography and other radiological techniques [1]. The non-specific symptoms and lack of efficient diagnostic methods, coupled with lower awareness among some clinicians, contribute to its underdiagnosis and misdiagnosis, resulting in a mortality of 2–11% at 5 years and 14% at 15 years. In addition, 3–13% of patients require interventional therapy to alleviate severe limb impairments [2]. Furthermore, TAK creates a substantial economic burden, particularly in terms of increased inpatient costs. A study found that patients with TAK had a mean additional $11,275 (95% confidence interval [CI]: $4946 to $17,603) in total hospital charges and a mean additional $45,305 (95% CI: $23,063 to $67,546) in total hospitalization charges compared to patients with TAK [3].

Despite decades since its discovery in the 1950s, the precise mechanisms of TAK remain extremely limited. The prevailing view suggests that TAK is an autoimmune disease where an unknown trigger initiates an immune response, leading to inflammation and the damage of arteries, tissues, and cells and causing the chemotaxis of macrophages and proliferation of fibroblasts and granulomas formation in the blood vessel walls. This leads to vascular stenosis, dilation, and even occlusion in large and medium-sized arteries, resulting in corresponding clinical symptoms [4].

Available data have shown some connection between factors and TAK, such as *Mycobacterium tuberculosis* infection or genetic susceptibility; however, the exact predisposing or detailed etiological factors are controversial, which makes the prevention and control of the disease a huge challenge. According to population surveys, the incidence of TAK in Asia shows an increasing trend [5]. It is estimated that in 2017, there were 5320 cases in Japan (95% CI: 4810–5820), whereas in 1994, this number did not exceed 5000 [6, 7]. Treatment involves both surgical and pharmacological interventions. For patients with emergency and severe disease, endovascular and surgical interventions can be used to treat patients, aiming for rapid symptom alleviation; however, surgical intervention alone does not significantly improve prognosis. Glucocorticoids (GCs) and methotrexate (MTX) are cornerstone therapies for the management of TAK. More recently, immunotherapy drugs such as tocilizumab have emerged due to a better understanding of the inflammatory mechanisms involved in TAK [1].

The NOD-like receptor family, pyrin domain containing 3 (NLRP3) inflammasome is the most extensively studied inflammasome. It is widely involved in various vascular diseases including atherosclerosis, Kawasaki disease, and anti-neutrophil cytoplasmic antibody-associated vasculitis [8]. Unfortunately, there are limited studies on its relationship with TAK. Recent studies have suggested that the NLRP3 inflammasome and the upstream priming and activation pathways are associated with the severity of TAK [9]. However, the precise underlying mechanisms remain unknown. Herein, this review summarizes the features of TAK as well as the relationship between its pathogenesis and targeting to the NLRP3 inflammasome (Table 1).

**Table 1.** **Diseases in different systems or organs related to the NLRP3 inflammasome**.

Organ/System	Related diseases
Cardiovascular System	CHD, Hypertensive cardiomyopathy, AF, VTE, Ischemic cardiomyopathy, Viral myocarditis, Dilated cardiomyopathy, Diabetic cardiomyopathy, Chemo/radiation-induced myocardial injury, Pericarditis, Vasculitis
Respiratory System	COPD, Allergic asthma, COVID-19, Allergic rhinitis, Chronic rhinosinusitis, ALI, Lung cancer, TB, Silicosis, PH, CF
Digestive System	*Helicobacter pylori* infection, Gastritis, NAFLD, Liver cirrhosis, CHB, DILI, Gastric cancer, Liver cancer, IBD, Chronic pancreatitis, Acute pancreatitis, Pancreatic cancer, CHC, AIH, PBC, PSC
Nervous System & Psychiatric Diseases	Ischemic stroke, AD, Epilepsy, PD, Hemorrhagic stroke, MS, HD
Metabolic System	Obesity, T2DM
Kidney Diseases	AKI, DN, Hypertensive nephropathy, LN
Connective Tissue & Autoimmune Diseases	RA, Gout, Psoriasis, Atopic dermatitis, CAPS
Hematological Diseases	NHL, MM, AML, Lymphoid Leukemia, MDS, GVHD, SCA
Infectious Diseases	Sepsis
Musculoskeletal System	IVDD
Oral Diseases	Periodontitis
Ophthalmic Diseases	Allergic conjunctivitis
Obstetric Diseases	Preeclampsia

AD, Alzheimer’s disease; AF, Atrial fibrillation; AIH, Autoimmune hepatitis; AKI, Acute kidney injury; ALI, Acute lung injury; ALL, Acute lymphoblastic leukemia; AML, Acute myeloid leukemia; CAPS, Cryopyrin-associated periodic syndrome; CF, Cystic fibrosis; CHB, Chronic hepatitis B; CHC, Chronic hepatitis C; CHD, Coronary heart disease; CMP, Cardiomyopathy; COPD, Chronic obstructive pulmonary disease; COVID-19, coronavirus disease 2019; DAMPs, Damage-associated molecular patterns; DILI, Drug-induced liver injury; DN, Diabetic nephropathy; GVHD, Graft-versus-host disease; HD, Huntington’s disease; IBD, Inflammatory bowel disease; IVDD, Intervertebral disc degeneration; LN, Lupus nephritis; MDS, Myelodysplastic neoplasms; MM, Multiple myeloma; MS, Multiple sclerosis; NAFLD, Non-alcoholic fatty liver disease; NHL, Non-Hodgkin lymphoma; NLRP3, NOD-like receptor family, pyrin domain containing 3; PAMPs, Pathogen-associated molecular patterns; PBC, Primary biliary cholangitis; PD, Parkinson’s disease; PH, Pulmonary hypertension; PSC, Primary sclerosing cholangitis; RA, Rheumatoid arthritis; SCA, Sickle cell anemia; T2DM, Type 2 diabetes mellitus; TB, Tuberculosis; VTE, Venous thromboembolism.

## 2. Methods

We conducted a narrative review of the English literature from PubMed, Web of Science, and Google Scholar databases without strict time restrictions for eligible publications. The reviewed literature primarily included human clinical or cellular studies, supplemented with animal experiments investigating relevant underlying mechanisms. We also incorporated retrospective studies, systematic reviews, meta-analyses, and narrative reviews. The search utilized the following keywords: NLRP3 inflammasome, Takayasu arteritis, vasculitis, epidemiology, incidence, prevalence, burden, pathology, clinical manifestations, pathogenesis, genetics, genes, inflammation, priming signal, activation signal, Toll-like receptors (TLRs), interleukins (ILs), IL-1β, IL-6, IL-18, heat shock proteins (HSPs), *M. tuberculosis*, Janus kinase (JAK), nuclear factor kappa B (NF-κB), signal transducer and activator of transcription 3 (STAT3), tumor necrosis factor alpha (TNF-α), reactive oxygen species (ROS), thioredoxin-interacting protein (TXNIP), mitophagy, P2X7 receptor (P2X7R), infliximab, etanercept, tocilizumab, and ziltivekimab.

## 3. Epidemiological, Pathological, and Clinical Features of TAK

TAK demonstrates significant epidemiological differences based on ethnicity and region. Previous studies have shown that the incidence and prevalence of TAK are higher in Asian populations, and the majority of patients are young women [5, 10, 11, 12]. A systematic review and meta-analysis including global studies showed a global average incidence of 1.11 (95% CI: 0.70–1.76) cases per million person-years, with a higher prevalence in Asia. The disease is more common in females, with an incidence of 2.01 (95% CI: 1.39–2.90) cases per million person-years (Table 2, Ref. [5, 6, 10, 12]) [13].

**Table 2.** **Brief summary of epidemiology research data on TAK**.

Area	Year	Total population size	Number of cases	Mean annual incidence cases per million (95% CI)	Prevalence cases per million (95% CI)	Age	Male to female ratio	Other findings	Reference number
China	2015 to 2017	14,429,700 in 2015,	102 prevalent cases and 68 incident cases.	2016 to 2017: 2.33 (1.97–3.21)	2015 to 2017: 7.01 (5.65–8.37)	Average patient age was 44 years. 16- to 34-year-old subgroup had a prevalence of 11.59 per million and incidence of 3.55 per million.	1:1.78	Type V was the most common (38.2%), followed by type I (22.6%), type IIa, (9.8%), type IIb (10.8%), type IV (11.8%), and type III (6.8%).	[5]
		14,660,000 in 2016,							
		14,551,300 in 2017.							
Japan	2017	Patients with records from 14,291 facilities in Japan.	2369	NR	NR	Median age at onset was 29 years.	1:1.77	Adult patients had a higher rate (57.8%) of comorbidity than young people (44.9%), with aortic regurgitation being the most common (35.0%).	[6]
								The affected rate of each artery: common carotid artery and internal carotid artery (62.7%), subclavian artery (57.8%), arch of aorta, (49.8%), and thoracic descending aorta (40.0%).	
US	2010 to 2018	Patients with records resided in 27 counties in the US.	5 prevalent cases and 1 incident case.	NR	2010 to 2015: 8.4 (1–15.8)	Mean age at diagnosis was 20.5 years.	1:3.61	Type V was the most common (80.0%), followed by type I (20.0%).	[10]
UK	2000 to 2005	About 3.6 million patients and 445,000 patients from 2 different databases.	16 prevalent cases and 14 incident cases from the UKGPRD.	0.8 (0.4–1.3)	7.1 (NR)	Median age at diagnosis was 51.0 years.	1:13.00	The incidence of TAK in the UK is similar to that in other countries.	[12]

CI, Confidence interval; NR, Not reported; TAK, Takayasu’s arteritis; UKGPRD, UK General Practice Research Database.

TAK can be divided into two main phases: active (systemic/acute) phase and inactive phase. In the initial stage of the active phase, inflammation primarily affects the vasa vasorum and the junction between the media and adventitia. Mononuclear cell infiltration, cellular edema, elastic fiber rupture, granuloma formation, and necrosis of the media can be observed. In the developing stage, there is reactive fibroplasia in the intima, and neovascularization occurs between the intima and media. Severe inflammatory reactions can induce the death of smooth muscle cells in the media, leading to gradual dilation of the vessels and even the formation of aneurysms. After entering the inactive phase, fibrosis and scar formation occur in the adventitia, and the infiltrating inflammatory cells mainly consist of plasma cells and multinucleated giant cells. Fibroplasia and granuloma formation compress the lumen, resulting in stenosis and the appearance of symptoms [14].

**Fig. 1.**
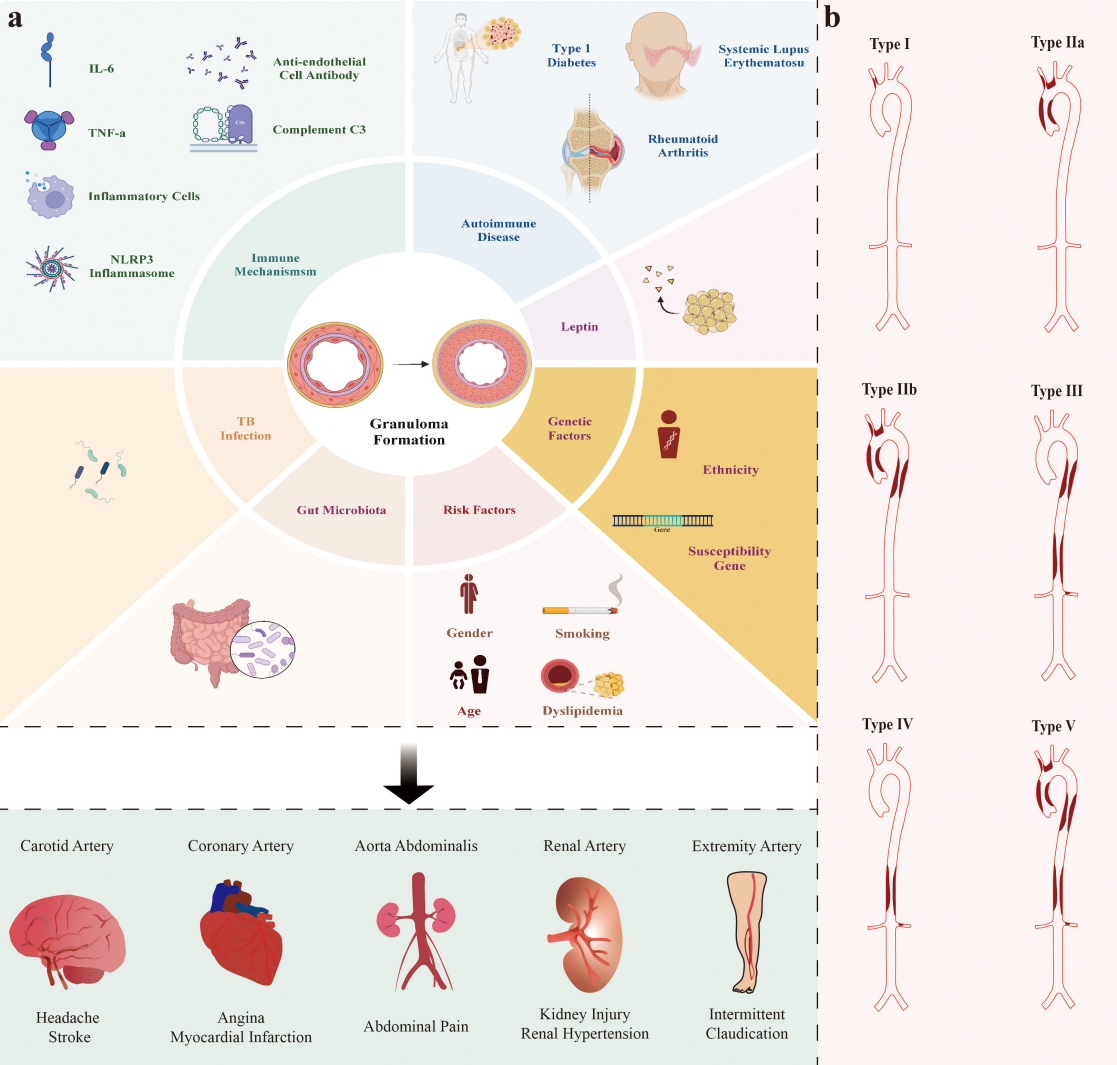
**Risk factors and potential pathogenesis/mechanisms involved in TAK and Hata’s angiografic classification of TAK**. (a) The risk factors and elements involved in the pathogenesis of TAK are not yet fully understood. Studies generally suggest that risk factors for TAK include sex (possibly related to estrogen levels), age, dyslipidemia, and smoking [23, 24, 25]. The pathogenesis of TAK involves multiple aspects such as genetics, immunity, and *M. tuberculosis* infection. Recent studies indicate that the pathogenesis of TAK may also be associated with new factors such as the gut microbiome and leptin level [26, 27]. It is also thought that TAK itself is an autoimmune disease, indicating a common underlying mechanism. In summary, different arterial involvements cause corresponding symptoms. (b) Hata’s classification is widely used in clinical practice to categorize TAK based on vessel involvement, which includes aortic arch, ascending aorta, thoracic descending aorta, abdominal aorta, renal arteries and their branches [28].

TAK can have an insidious onset or present acutely, which manifests as acute ischemic necrosis such as stroke or myocardial infarction [15]. Symptoms depend on which blood vessels are affected. When the renal arteries are involved, hypertension can occur. Severe narrowing or occlusion of the blood vessels in a short period of time can lead to a hypertensive crisis. This sudden and significant increase in blood pressure can cause various symptoms such as headache and nausea [16, 17]. Aortic valve regurgitation is a significant complication of TAK and can lead to heart failure, which is a main cause of death in patients with TAK [18]. TAK involvement of the coronary arteries is rare, with an incidence of 6–30% in patients and a 3% chance of causing acute myocardial infarction [19, 20]. TAK is a cause of renal impairment in children and can also contribute to growth retardation [21, 22]. Increased erythrocyte sedimentation rate (ESR) and C-reactive protein (CRP) are commonly observed in patients with TAK. However, it is noteworthy that only less than 20% of patients show an increased white blood cell count (Fig. 1, Ref. [23, 24, 25, 26, 27, 28]) [29].

For stenosis, vascular intervention treatment is considered the routine option; however, patients with TAK are prone to restenosis after this intervention due to the persistent inflammatory response inherent in this disease. The in-stent restenosis rate is as high as 60% in patients with TAK, but only 5–10% in patients with coronary heart disease [30, 31]. Clinicians should be aware of the aforementioned complications associated with TAK and the importance of combined anti-inflammatory treatment, as routine interventional therapy often yields poor results.

## 4. Traditional Risk Factors and Related Genes for TAK

The current pathogenesis theory suggests that the persistent inflammatory response leading to TAK may be caused by microbial antigens [32]. Increasing studies point to a new understanding that the vascular system may not be completely sterile, and immune reactions to pathogenic protein antigens may cross-react with natural proteins on host vascular tissue cells [33]. Kumar Chauhan S *et al*. [34] found elevated levels of HSP65 in the arterial tissue of patients with TAK. HSP65 is found in pathogens such as *M. tuberculosis* and is highly similar to HSP60, which is widely present in human tissue cells. Patients with TAK develop specific antibodies recognizing both HSP60 and HSP65 [26]. Antigens produced by pathogens, such as lipopolysaccharide (LPS), may be recognized by antigen-presenting cells (APCs) and the pathogen’s exotoxins can upregulate the expression of TLRs on APCs and mononuclear-macrophages. Activation of T cells, B cells, and natural killer (NK) cells induces the apoptosis of cells through various pathways. Additionally, HSP65 stimulates arterial tissue cells to synthesize MHC class I polypeptide-related sequence A proteins, which bind to NKG2D receptors on γδ T cells or NK cells, promoting the secretion of perforin and causing cell apoptosis. T-cell activation and vessel tissue cell death induce the release of inflammatory factors such as TNF-α, which continuously stimulate mononuclear macrophage aggregation and fusion into giant cells that secrete matrix metalloproteinases and TNF-α. The persistent activation of immune cells and fibroblasts leads to vascular tissue fibrosis and granuloma formation. IL-6, primarily released by NK cells, monocytes, and vascular endothelial cells, promotes fibrosis and plays an important role in persistent inflammatory reactions [35]. Inhibiting the IL-6 receptor (IL-6R) can effectively improve the symptoms of TAK [36]. In addition, self-antibodies targeting endothelial protein C receptor and scavenger receptor class B type 1 have been discovered in patients with TAK, which disrupt the normal function of vessel well, leading to an imbalance in the inflammatory response and potential cell death [37].

Studies suggest that TAK has genetic factors influencing susceptibility and is prevalent in individuals of Asian and Slavic descent, including those who have immigrated to Western countries, compared to the local white population [12, 38]. The *HLA-B52* gene is the most well-known related genetic factor, and is more prevalent in high-incidence Asian populations compared to European populations. Meanwhile other newly discovered risk factors include sushi, von Willebrand factor type A, epidermal growth factor (EGF) and pentraxin domain containing 1, cofilin 2, *VPS8*, *chr13q21*, and the *IL18-137G/C* polymorphism, while protective factors include the *IL18-607C/A* polymorphism and killer cell immunoglobulin-like receptor *2DS4* gene; however, further studies are needed to determine the ethnic distribution of these genes (Fig. 1) [39, 40, 41, 42, 43, 44, 45, 46].

## 5. Role of NLRP3 Inflammasome in the Development of TAK

The activation of inflammasomes is closely related to many diseases with an inflammatory component. The inflammasome causes cell pyroptosis and participates in the innate immune response, triggering local as well as systemic inflammatory responses. Activation of the inflammasome occurs by the binding of cysteinyl aspartate specific proteinase 1 precursor (pro-caspase 1) to pattern recognition receptors (PRRs) through apoptosis-associated spot-like protein (ASC). PRRs, particularly the NOD-like receptor family (e.g., NLR family pyrin domain containing 1 [NLRP1], NLRP2, NLRP3, NLRP6, NLRP12, NLR family caspase recruitment domain (CARD) domain-containing protein 4 [NLRC4]), are key players in the immune system’s ability to detect and respond to threats. PRR can initiate pathways that lead to the activation of pro-caspase-1 and its subsequent regulation of IL-1β and gasdermin D (GSDMD). After hydrolysis, gasdermin D N-terminal fragment (GSDMD-NT) causes cell membrane perforation and initiates pyroptosis. Cell membrane perforation leads to the release of IL-1β and IL-18, which subsequently aggravate the inflammatory response. The recognition of pathogen-associated molecular patterns (PAMPs) and damage-associated molecular patterns (DAMPs) by PRRs, such as TLRs, triggers a cascade of events. These events involve the activation of signaling pathways such as NF-κB and interferon regulatory factors, causing the transcriptional upregulation of inflammatory mediators. Ultimately, this results in the activation of inflammasomes, which amplify the inflammatory response through the maturation of cytokines and induction of pyroptosis [47]. NLRP3 inflammasome activation involves three pathways: the ATP-mediated pathway, lysosome-related pathway activated by cathepsins, and the mitochondria-dependent pathway mediated by oxidative stress. However, the specific molecular mechanisms of inflammasome activation and assembly are not fully understood [48, 49].

### 5.1 Priming and Activation Signals of NLRP3 Inflammasome in TAK

The canonical pathway for NLRP3 inflammasome activation generally requires two distinct signals: a priming signal and an activation signal. The priming signal (signal I), involves the upregulation of NLRP3 and pro-IL-1β, typically triggered by NF-κB activation via PRRs like TLRs or cytokine receptors. The activation signal (signal II) triggers the assembly of the inflammasome complex, often involving substances like double-stranded deoxyribonucleic acid (dsDNA), double-stranded ribonucleic acid (dsRNA), extracellular adenosine triphosphate (eATP), and potassium ions. Thus, the NLRP3 inflammasome is activated through a series of events rather than a direct interaction [50].

There is evidence supporting the idea that HSP65 is highly expressed in vascular tissues and may act as a priming signal as a TLR agonist [51]. It is a member of the HSP60 family, mainly presenting in *M. tuberculosis* and originally isolated from *My. bovis*. HSP65 shares a high degree of similarity with human HSP60 [52]. Additionally, HSP60 can activate macrophages by interacting with TLRs, initiating the synthesis of NLRP3 and triggering inflammatory cascades [53]. Huang *et al*. [54] found that in patients with gouty arthritis, HSP60 expression was increased in peripheral blood mononuclear cells (PBMCs) and HSP60 activated the TLR4/myeloid differentiation primary response protein 88 (MyD88)/NF-κB signaling pathway upon binding to TLR4, resulting in the synthesis of the NLRP3 inflammasome and contributing to mitochondrial dysfunction. Naturally, there is a question of whether HSP65 also possesses the same function, and there are some clues supporting this idea. First, scholars replicated animal models of gouty arthritis by injecting inactivated bacillus Calmette-Guerin vaccine because HSP65 can serve as an antigen in synovial tissue and shares a conserved sequence with HSP60 [55]. Moreover, Bulut *et al*. [56] summarized the mechanisms by which HSP65 and HSP70 induce inflammation through studies of immune processes in *M. tuberculosis* infection. The authors found that HSP65 can activate NF-κB by stimulating TLR4 with or without MyD88 protein and promote inflammation [56]. However, an earlier study showed that HSP65 has weak binding affinity to TLR4 in the presence of cluster of differentiation 14 and myeloid differentiation factor 2, and that HSP65 cannot activate NF-κB through the TLR4-mediated pathway [57]. In addition, Wu *et al*. [58] found that HSP65 upregulates the expression of IL-6 and IL-1β in aorta adventitial fibroblasts through the TLR4/JAK2/AKT/STAT3 pathway *in vitro*. Based on the information available, there is no direct evidence that HSP65 directly initiates the synthesis of the NLRP3 inflammasomes. However, there is evidence that HSP65 can trigger the production of pro-inflammatory cytokines, suggesting a role in these immune responses.

TNF-α plays a key role in the pathogenesis of TAK. The levels of TNF-α in the peripheral blood and lesion tissues of patients with TAK are significantly higher than those of healthy individuals. TNF-α can bind to the TNF receptor and send a priming signal to promote the upregulation of NLRP3 [59]. Studies have shown that the use of TNF-α inhibitors can alleviate the activity of TAK, which we will describe in detail later.

According to current understanding, activation signals may involve multiple factors. After cell perforation, DNA fragments, specifically extracellular dsDNA (eDNA), are released from cells undergoing death due to the activity of γδ T cells or NK cells and are engulfed by macrophages. Within these macrophages, the engulfed DNA fragments can activate the NLRP3 receptor, which in turn leads to the activation of caspase-1, ultimately resulting in the formation of the NLRP3 inflammasome. Inflammasomes activation leads to the production of inflammatory factors such as IL-1 and IL-18, as well as the activation of GSDMD, resulting in pyroptosis. Inflammatory factors and eDNA released after pyroptosis then initiate the next cycle [60].

Also, an alternative activation pathway of NLRP3 inflammasomes exists in human monocytes. The binding between LPS and TLR4 can stimulate the assembly of NLRP3 inflammasomes, and this can occur through a pathway involving receptor-interacting serine/threonine-protein kinase 1, Fas-mediated death domain protein, and caspase-8, bypassing the priming step [61]. It has also been found that TLR4 recognizes LPS in the PBMCs of patients with TAK, which increases the expression of IL-1β and IL-1R2 [62]. LPS can independently trigger assembly of the NLRP3 inflammasome and increase the level of IL-1β in the absence of a priming signal in macrophages [63].

### 5.2 ROS-TXNIP-NLRP3 Axis and TAK

In 2014, Maejima *et al*. [9] found a correlation between the severity of symptoms in patients with TAK and polymorphism of the MAX Dimerization Protein MLX (*MLX*) gene. Specifically, patients with TAK with severe symptoms had a higher frequency of the missense mutation *rs665268* in the *MLX* gene, indicating a potential association between MLX protein and TAK pathogenesis [9]. Previous studies have established a correlation among MLX protein, TXNIP, and NLRP3 inflammasome activation, particularly under conditions of oxidative stress and involving the mitochondria [64, 65]. Research suggests a link between *MLX* allelic variants and the NLRP3 inflammasome, which plays a role in the pathogenesis of TAK. In 2014, a study by Maejima *et al*. [9] confirmed that the *MLX* risk genotype upregulates MLX protein transcription, which in turn promotes the expression of TXNIP through the MLX/Mondo-A complex. The authors also observed an increase in NLRP3 inflammasome and caspase-1 levels in the cultured macrophages of patients with TAK harboring this allele compared to the control group [9].

Additionally, this genotype was associated with decreased autophagy levels, possibly due to reduced free Mondo-A resulting from its binding to the MLX protein. Mondo A promotes autophagy by inhibiting the expression of Rubicon, which in turn acts as a brake on autophagy, particularly by inhibiting the formation of auto-phagolysosomes (autophagosomes fused with lysosomes for degradation). Rubicon accomplishes this by binding to the phosphoinositide 3-kinase complex and inhibiting its activity [66].

Mitophagy plays a crucial role in the negative regulation of the NLRP3 inflammasome by clearing damaged mitochondria, one of the sites that generate upstream activation signals for NLRP3. Damaged mitochondria release ROS and oxidized mitochondrial DNA into the cytosol, which induce activation of the NLRP3 inflammasome through mechanisms that are not fully understood [67]. Moreover, the NLRP3 inflammasome can be engulfed by autophagosomes through ubiquitination and subsequent degradation. Ubiquitination can activate autophagy, resulting in the inactivation of NLRP3 inflammasomes [68].

Macro-autophagy and ROS act as intermediaries in the correlation between STAT3 and NLRP3. Phosphorylated STAT3 acts as a transcriptional activator, binding to target genes and promoting their expression. In mice, activation of STAT3 increases the acetylation of histones H3 and H4 on the NLRP3 promoter, thereby promoting the expression of effector proteins. Applying JAK inhibitors to reduce STAT3 phosphorylation will continuously inhibit NLRP3 transcription, suppressing inflammasome expression [69]. In addition, stimulating the marrow-derived macrophages of mice with LPS showed that STAT3 inactivation blocked NLRP3 inflammasome-induced caspase-1 activation and IL-1β secretion and STAT3 downregulated mitochondrial autophagy mediated by PTEN-induced kinase 1 (PINK1), positively regulating macrophage NLRP3 inflammasome activation [70].

### 5.3 NLRP3 and P2X7R in TAK

One of the activation pathways of NLRP3 inflammasome is mediated by the P2X7R starting with signal II [47, 71]. P2X7R is a transmembrane receptor, and ATP is its only physiological agonist [72]. It is widely expressed in various cells of the human body including endothelial cells, fibroblasts, neutrophils, monocytes/macrophages, dendritic cells, and lymphocytes [73]. Binding of the P2X7R with its agonist causes potassium efflux, sodium and calcium influx, and the formation of non-selective pores that allow the passage of macromolecules [74]. Cell damage or death leads to the release of ATP, which activates the P2X7R, resulting in the variation of these electrolytes and the entry of PAMPs through the pores [75, 76]. These actions disrupt the cellular homeostasis, leading to organelle damage, ROS release, initiation of the ROS-NLRP3 axis, and assembly of the NLRP3 inflammasome [77]. P2X7R can also mediate the release of IL-1β and IL-18, inhibit the differentiation of regulatory T cells, induce the conversion of T helper 17 cells (Th17 cells), and promote inflammatory responses [78, 79].

Monocytes/macrophages with high expression of P2X7R are found in the vascular wall [80]. P2X7 polymorphisms are associated with both TAK and tuberculosis infection. The *P2X7-1513C* is a mutation that leads to non-functional P2X7R expression in macrophages. The frequency of this polymorphism is higher in patients with TAK than those with only pulmonary tuberculosis, and tuberculosis genetic material can also be detected in the aortic arch and other arterial tissues of patients with TAK [81]. Theoretically, the extent of pyroptosis mediated by the eATP-P2X7R pathway in the macrophages of patients with TAK would be weakened according to this finding, which would partially compromise the innate immunity against *M. tuberculosis*. It has been hypothesized that the proliferation of *M. tuberculosis* through the aforementioned mechanism can lead to the increased synthesis of HSP65 protein and exacerbate TAK.

### 5.4 NLRP3, ILs, and TAK

Cells release two main inflammatory mediators, IL-1 and IL-18, after NLRP3 inflammasome activation. IL-1 has strong inflammatory-inducing effects that can lead to prostaglandin secretion and activate and differentiate neutrophils, monocytes, and lymphocytes [82]. IL-18 is a key player in inducing IFN-γ production in macrophages and NK cells, as well as increasing the synthesis of TNF-α and IL-2 [79]. The high expression of the IL-1β gene has been observed in patients with TAK, and a polymorphism in the *IL-1β* gene is associated with TAK susceptibility [62, 83]. In addition, plasma levels of IL-18 are significantly elevated in patients with TAK, and a polymorphism in the promoter region of the *IL-18* gene is correlated with TAK susceptibility. There are three tag single nucleotide polymorphisms, namely, *IL18-656G/T (rs1946518)*, *IL18-607C/A (rs1946519)*, and *IL18-137G/C (rs187238)* [84]. Among them, *IL18-607C/A* is a protective factor that may reduce the risk, whereas *IL18-137G/C* and *IL18-656G/T* are considered risk factors that may increase the risk of TAK. The *IL18-137G/C* and *IL18-656G/T* polymorphisms are associated with higher IL-18 levels [44, 46].

As aforementioned, there is a close relationship between IL-6 and TAK. The levels of IL-6 in the peripheral blood and lesion tissues of patients with TAK are significantly elevated compared to healthy individuals, and IL-6 is significantly involved in the pathogenesis of TAK. First, IL-6 is closely associated with changes in inflammatory markers such as ESR and CRP, which can reflect the severity of inflammation during the acute phase of the disease. Second, in the early stage of TAK, monocytes and fibroblasts are activated through the TLR/NF-κB pathway, and the recognition of PAMPs induces the expression of IL-6. In other words, the level of IL-6 can reflect the expression level of TLRs. Therefore, IL-6 may be a sensitive indicator in the early stage of the disease. Lastly, the high level of IL-6 is also prominent among cytokines during the fibroproliferative phase. IL-6 promotes the proliferation of lymphocytes and is secreted by lymphocytes, as well as macrophages, epithelial cells, and fibroblasts [85]. In conclusion, IL-6 is closely related to the inflammation, fibroproliferation, and granuloma formation observed in TAK [86, 87].

Researchers have observed in a mouse model of rheumatoid arthritis (RA) that IL-6 can activate the NLRP3 inflammasome in the presence of ATP. Conversely, blocking the IL-6 pathway leads to reduced NLRP3 inflammasome [62]. The two major downstream signaling pathways activated by IL-6/IL-6R are JAK/STAT3 and JAK/mitogen-activated protein kinase. Studies have shown that IL-6 may also influence the initiation of NLRP3 inflammasome mediated by NF-κB. Dai *et al*. [88] knocked in human IL-6R genes into mice and induced them to express, and then synthesized antibodies against these receptors to block their effects. The results showed that the NF-κB signaling pathway was also affected in specific cells, and the initiation and activation of the NLRP3 inflammasome, IL-1β secretion, pro-caspase-1 cleavage, and GSDMD activation were all inhibited [88]. Researchers showed that certain herbal extracts used to treat ulcerative colitis demonstrated dual effects by blocking the IL-6/JAK2/STAT pathway and also reducing ASC protein expression and NLRP3 inflammasome formation. They interpreted this phenomenon as double inhibition of two distinct pathways involved in inflammation. However, combined with previous findings, this may be due to blocking the upregulation of ASC protein expression mediated by IL-6, thereby affecting the NLRP3 inflammasome [89]. Furthermore, IL-6/JAK/STAT3 pathway activation can lead to inhibition of mitophagy, increased ROS release, and promotion of inflammasome activation, a process that is dependent on PINK1 [90].

Research on lung tissues infected with SAR-CoV-2 has revealed a cascade reaction of NLRP3/IL-1β/IL-6 in patients with coronavirus disease 2019 (COVID-19). The secretion of IL-1β related to NLRP3 inflammasome-mediated pyroptosis can promote the release of IL-6 by leukocytes, exacerbating the condition of COVID-19 [91]. Sonowal *et al*. [92] showed that luxeptinib can inhibit the hydrolysis of caspase-1 and NLRP3-mediated IL-1β release. It also inhibits the release of TNF-α and IL-6 by influencing other inflammatory signaling pathways, including those activated independently of the NLRP3 inflammasome. Luxeptinib directly affects IL-6 and TNF-α release through these distinct pathways, not solely via its impact on IL-1β [92]. This suggests that IL-6 is a downstream factor of IL-1β regulated by the NLRP3 inflammasome, and seems to affect the levels of IL-6 through IL-1β, suggesting the existence of a positive feedback regulation [58]. In conclusion, NLRP3 and ILs are intricately linked and play a crucial role in inflammation, with complex signaling pathways like those involving TAK.

## 6. TAK, NLRP3 Inflammasome, and NETosis

The inflammation response in TAK involves a complex interplay between neutrophil extracellular traps (NETs) and NLRP3 inflammasome activation. NETosis is a process in which neutrophils, upon external stimulation, undergo disintegration of nuclear membranes and decondensation of chromatin structures to form fibrous extracellular networks called NETs. These NETs are composed of DNA, citrullinated histone H3 (CitH3), and other proteins, which are subsequently released into the extracellular space. NETs play a dual role in the immune defense by trapping, containing, and even killing pathogens in the extracellular environment, while simultaneously exacerbating inflammatory responses [93].

Suicidal NETosis, one form of NETosis, is triggered by stimuli such as IL-6, IL-8, TNF-α, and pathogens. Upon binding to their corresponding receptors like TLRs, these stimuli activate nicotinamide adenine dinucleotide phosphate (NADPH) reduced form oxidase to generate ROS. ROS then trigger the release of myeloperoxidase (MPO) and neutrophil elastase (NE) from cytoplasmic granules. With the assistance of peptidylarginine deiminase 4, NE, and MPO mediate chromatin decondensation and membrane rupture, ultimately leading to the release of NETs [94, 95].

In patients with TAK, researchers have observed that NET levels correlate with disease activity, and serum samples exhibit the presence of anti-citrullinated protein/peptide antibodies (ACPA) targeting CitH3 [96]. In other diseases or animal models, factors implicated in TAK pathogenesis such as IL-6, TNF-α, LPS, and *M. tuberculosis* induce NET formation [97, 98]. Notably, HSP60, an important component linking to TAK pathogenesis, can be targeted by altered peptide ligands (APLs). These APLs competitively bind receptors and exert immunomodulatory effects to attenuate HSP60-driven pathology. In experimental models of RA and other conditions, APLs reduce NET formation, suggesting a connection between HSP60 and NETosis, which may serve as a bridge linking NETosis to TAK as well [99]. Additionally, NETs may exacerbate TAK inflammation by promoting CitH3-induced ACPA production and activating complement C3, although direct evidence for this mechanism is lacking [100].

Activation of the NLRP3 inflammasome facilitates the production of NETs. The formation of inflammasome-associated ASC is observed prior to chromatin decondensation, and the presence of ASC and caspase-1 associated with CitH3 is also noted in NETs formed in other specific diseases [101]. In the disease model of gout, the absence of Caspase-11, downstream of the non-canonical NLRP3 pathway, restricts the generation of neutrophil NETs, whereas which should formed under the stimulation of monosodium urate (MSU) [102]. IL-1β, potentially derived from NLRP3 inflammasome-mediated pyroptosis, not only exacerbates the NETosis process but also induces IL-1β production in bronchial tissue cells exposed to NETs, suggesting a possible interrelationship between NLRP3 inflammasome activation and NETosis [103]. It is hypothesized that the formation of NETs may be related to the NLRP3-Caspase 1/caspase 11-GSDMD pathway, and the chromatin components within NETs might also stimulate the synthesis of the NLRP3 inflammasome.

In summary, TAK involves both NETosis and NLRP3 inflammasome activation, with NLRP3-mediated regulation to NETosis observed in other diseases. However, no studies to date have directly determined whether neutrophil-derived NETs and NLRP3 inflammasome activation co-occur in TAK. Analyzing their interplay faces challenges due to the disease’s complex and unclear mechanisms, making it difficult to determine the temporal sequence between NETosis and NLRP3 activation. Moreover, numerous mechanistic questions remain unresolved, such as whether NET components like CitH3 and dsDNA trigger NLRP3 assembly or whether GSDMD-mediated pyroptosis amplifies NETosis in TAK. Further research is needed to elucidate these interactions and their contribution to vascular inflammation.

## 7. Pharmacotherapies Targeting NLRP3 in Patients With TAK

The pharmacotherapy used to treat TAK includes using GCs, immunosuppressive agents, and newer targeted biologic drugs like tocilizumab and infliximab [1, 104]. GCs are well-known anti-inflammatory agents and are recommended by the American College of Rheumatology (ACR) as first-line therapy for TAK [1]. Using GCs alone can achieve clinical remission for 60% of patients, but relapse is common after dosage reduction [105]. Studies have found that GCs can inhibit the activity of NLRP3 inflammasomes, reducing levels of IL-1β and IL-18. Pretreating macrophages that have not yet developed inflammation with GCs can block the effect of NF-κB triggered by signal I, while GCs can also affect signal II and inhibit the production of mitochondrial ROS and NLRP3 within macrophages that have already been exposed to PAMPs. However, few investigations have been directly conducted in patients with TAK, and the specific mechanism by which GCs affect NLRP3 inflammasome in TAK remains unclear [106, 107].

**Fig. 2.**
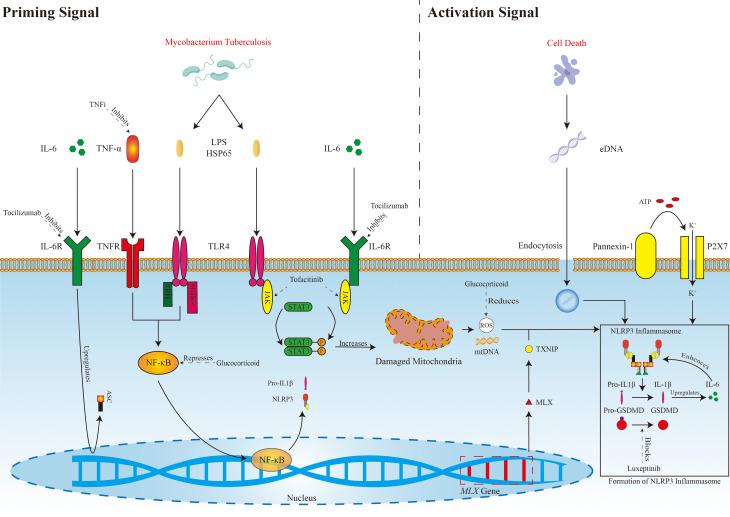
**Possible mechanisms of NLRP3 inflammasome activation in TAK and targeting pathways**. TNFi, Tumor necrosis factor alpha inhibitor; IL-6, Interleukin-6; TNF-α, Tumor necrosis factor-α; LPS, lipopolysaccharide; HSP65, Heat shock protein 65; eDNA, extracellular DNA; IL-6R, Interleukin-6 receptor; TNFR, Tumor necrosis factor receptor; TLR4, Toll-like receptor 4; ATP, Adenosine triphosphate; JAK, Janus Kinase; STAT3, Signal transducer and activator of transcription 3; ROS, Reactive oxygen species; mtDNA, mitochondrial DNA; NLRP3, NOD-like receptor pyrin domain-containing protein 3 receptor; IL-1β, Interleukin-1β; GSDMD, Gasdermin D; ASC, Apoptosis-associated spot-like protein; NF-κB, nuclear factor kappa B; MLX, MAX dimerization protein MLX.

Although GC therapy is a cornerstone treatment for some patients, some individuals either do not achieve full remission or experience relapses. In such cases, targeted drugs offer several advantages such as reduced hormone dosage, enhanced remission rates, and decreased recurrence. Major targets for targeted drug therapies in TAK include blocking TNF receptors, TLR4, IL-6Rs, and the JAK/STAT3 pathway (Fig. 2).

### 7.1 TNF-α Inhibitor

Infliximab is a TNF-α inhibitor, and TNF-α is produced by macrophages, T cells, and NK cells, promoting the proliferation of Th1 cells and playing a crucial role in granuloma formation. TNF-α can bind to TNF receptors and activate NF-κB, priming the synthesis of components of the inflammasome. NF-κB and NLRP3 also promote the upregulation of TNF-α [59, 108]. This feedback loop enhances the activity of NLRP3. The specific inhibition of TNF-α by infliximab blocks the aforementioned pathway and alleviates symptoms of TAK. Case reports and clinical trials have shown that infliximab may be effective in treating patients with refractory TAK and potentially reduces their dependence on GCs. Adalimumab is another type of TNF-α inhibitor that may be more efficacious than tocilizumab when combined with GCs and MTX for patients with active and severe TAK [109].

The formation of NLRP3 inflammasomes in TAK involves the canonical pathway mediated by a priming signal and activation signal. TNF-α and IL-6 released by tissue cells, or LPS and HSP65 released by *M. tuberculosis*, which after binding to their respective receptors, activate NF-κB, thereby enhancing the expression of NLRP3, ASC, pro-IL1β, and IL-6. LPS, HSP65, and IL-6 may also inhibit damaged mitochondrial clearance via the TLR4/JAK/STAT3 or IL-6/JAK/STAT3 pathway, leading to increased generation of ROS and mitochondrial DNA. TXNIP plays a crucial role in activation of the ROS pathway and its expression is facilitated by the MLX protein. There is a link among cell death, inflammasome activation, and the P2X7R in TAK. IL-1β activated by the NLRP3 inflammasome increases IL-6 expression, further enhancing the activation of the NLRP3 inflammasome itself in a positive feedback loop. GCs reduce the production of inflammasomes within the cells of patients with TAK by inhibiting NF-κB activity and ROS generation, whereas tocilizumab, TNF-α inhibitors, and tofacitinib block target proteins, reducing inflammasome production. Luxeptinib inhibits GSDMD cleavage and thus halts pyroptosis.

Etanercept is another type of TNF inhibitor used for the treatment of TAK; it blocks the effects of TNF-α and TNF-β by competitive inhibition. A prospective cohort study showed that anti-TNF-α therapy, such as Etanercept, as a succedaneum of infliximab in the trial, in combination with GCs, can be beneficial in treating patients with recurrent TAK, with 10 of 15 patients able to gradually reduce the dose of GCs or discontinue GC and achieve sustained remission [110]. Further research is needed to compare the respective advantages and disadvantages of Etanercept and infliximab in treating TAK.

### 7.2 IL-6 Inhibitor

Tocilizumab is an IL-6R monoclonal antibody, and the NLRP3 inflammasome may be a crucial component in TAK. Targeting this pathway with drugs like tocilizumab may be a relevant treatment strategy that could affect ASC synthesis, the clearance of damaged mitochondria, or even directly inhibit the function of NLRP3 by blocking the IL-6R, as discussed previously. Tocilizumab not only blocks the IL-6R but also inhibits activation of the NLRP3 inflammasome through the IL-17a pathway, leading to a significant decrease in IL-17a expression [111]. IL-6 promotes the differentiation and formation of Th17 cells, and the secretion of cytokines such as IL-17 and TNF-α [112]. Tocilizumab blocks the binding of IL-6R to IL-6, thereby weakening the secretion of IL-17 and its positive promotion of NLRP3 inflammasome. Numerous studies have observed the differentiation of CD4+ cells in the Th17 cells of patients with TAK, as well as increased levels of IL-17 in the arterial tissues and peripheral blood [113, 114]. Tocilizumab shows better efficacy in treating refractory TAK compared to traditional therapies. It also has a good safety profile but has raised concerns about rapid symptom relapse after discontinuation [115, 116]. The addition of tocilizumab in patients with poor GC response is recommended in ACR guideline [1]. There are also some targeted drugs that have not been sufficiently studied in clinical research, which may alleviate TAK and reduce recurrence.

Ziltivekimab is a novel IL-6 ligand human monoclonal antibody that alleviates inflammation and reduces serum CRP levels. It was developed to provide a new option for the anti-inflammatory treatment of atherosclerosis in patients with chronic kidney disease, as increasing data suggest that the pro-inflammatory effects of IL-6 can exacerbate atherosclerosis and thrombosis. Studies have found that regular use of this drug, with subcutaneous injections of 7.5, 15, or 30 mg every 4 weeks for 24 weeks, can effectively lower CRP levels in patients with significant inflammatory responses [117]. It is currently unknown whether ziltivekimab has a positive effect on cardiovascular outcomes, as clinical trial results are not yet available. To date, ziltivekimab has not been used for the treatment of TAK and its therapeutic efficacy remains to be substantiated.

### 7.3 TLR Inhibitor

TAK-242 (also known as Resatorvid) is a specific inhibitor of TLR4 signaling that shows promise for alleviating diseases such as ulcerative colitis [118, 119]. Wu *et al*. [58] used TAK-242 to block HSP65 activation of the TLR4-JAK2/AKT/STAT3 pathway and reduce the phosphorylation levels of JAK2, AKT, and STAT3 in aortic adventitial fibroblasts *in vitro*, showing a possibility as a targeted therapy. However, for treating other indications like sepsis, TAK-242 is either undergoing Phase 3 clinical trials or the trial results are unsatisfactory, limiting its clinical application.

### 7.4 JAK Inhibitor

JAK inhibitors work by blocking the JAK/STAT3 signaling pathway. Traditionally, it is believed that JAK inhibitors achieve therapeutic effects by reducing Th1- and Th17-derived cytokines and activating CD4+ cells. Therefore, JAK inhibitors may also influence the initiation and activation signals of the NLRP3 inflammasome through two mechanisms: blocking STAT3 upregulation of NLRP3 protein expression and enhancing the clearance of damaged mitochondria. Clinical trials have shown that the combined treatment with drugs such as tofacitinib and ruxolitinib for the treatment of TAK yields better results than using GCs alone [120, 121]. Currently, several clinical trials are underway (e.g., NCT04161898 and NCT04299971) (Table 3, Ref. [58, 109, 115, 120, 121, 122, 123, 124, 125, 126, 127]).

**Table 3.** **Recent studies targeting inflammasome-related treatments in TAK from 2012 to 2024**.

Reference	Target factor	Study design	Result	Reference number
Mertz *et al*.	TNF-α	Clinical trial	Infliximab could be an effective GC-sparing agent for TAK refractory to conventional therapy.	[122]
2020	IFX	23 patients from 12 centers	Rate of response after a median treatment duration of 36.9 months (IQR: 10.0–58.7): 64%	
		Median dose of GCs at initiation: 10 mg/day (IQR: 10–25).	GC dose decreased from median 10 mg/day (range 5–45 mg/day) at baseline to less than 10 mg/day at 12 months (*p * < 0.05).	
		Median dose of infliximab: 5 mg/kg (IQR: 5–5) every 6 weeks (IQR: 4–9).		
Mekinian *et al*.	TNF-α	Retrospective study	Infliximab may represent an interesting alternative therapeutic option even in refractory TAK.	[123]
2012	IFX	15 patients	Rate of response at 3 months: 87%	
		Median dose of GCs at initiation: 20 mg/day (range 5–35 mg/day).	Rate of response at 6 months: 77%	
			Rate of response at 12 months: 73%	
		Median dose of infliximab: 5 mg/kg (range 3–5 mg/kg) at a median of every 6 weeks (range 4–8 weeks).	Clinical and biological activities significantly decreased within 3 months (from 11 at baseline to 4 patients at 12 months; *p * < 0.05).	
			GC dose decreased from median 20 mg/day (range 5–35 mg/day) at baseline to median 6 mg/day (range 2.5–30 mg/day) at 12 months (*p * < 0.05).	
Misra *et al*.	IL-6 receptor and TNF-α	Systematic review and meta-analysis	Tocilizumab and TNFi had similar rates of clinical remission (RR [tocilizumab vs. TNFi]: 1.03, 95% CI 0.91–1.17), angiographic stabilization (RR 1.00, 95% CI 0.72–1.40) or adverse events (RR 0.84, 95% CI 0.54–1.31) based on observational data.	[124]
2023	TCZ and TNFi	6 studies and 491 patients were included.		
Kang *et al*.	IL-6 receptor	Systematic review and meta-analysis	Rate of decrease in GC dosage was 76% (95% CI 58–87%). Remission rate was 79% (95% CI 69–86%). Relapse rate was 17% (95% CI 5–45%). Imaging progress rate was 16% (95% CI 9–27%). Adverse events occurred in 16% (95% CI 5–39%) of patients, and infection was the most common adverse event, with a rate of 12% (95% CI 5–28%).	[125]
2023	TCZ	19 studies and 466 patients were included.		
Wang *et al*.	IL-6 receptor and TNF-α	RCT	ADA combined with GCs and MTX may be more efficacious than TCZ combined with GCs and MTX in patients with active and severe TAK.	[109]
2024	TCZ and ADA	40 patients were included.		
		ADA (*n* = 21) combined with GCs and MTX vs. TCZ (*n* = 19) combined with GCs and MTX.	ERs of the ADA group and TCZ group at 6 months were 85.71% (*p* = 0.02) and 52.63% (*p* = 0.02) in the ITT population, 89.47% (*p* = 0.06) and 62.50% (*p* = 0.06) in the PPS. The ERs at 9 and 12 months were similar (*p * > 0.05).	
Nakaoka *et al*.	IL-6 receptor	RCT	TCZ was superior to placebo for time to relapse of TAK and steroid-sparing effect without new safety concerns.	[115, 126]
2018 and 2020	TCZ	36 patients (18 received TCZ s.c. 162 mg/week and 18 received placebo after remission from TAK for ≥1 week) were enrolled in the double-blind period followed by the open-label extension period (36 received tocilizumab 162 mg/week s.c.) to evaluate the steroid-sparing effect of tocilizumab.		
			In the double-blind period, HRs for time to relapse were 0.41 (95.41% CI 0.15–1.10; *p* = 0.0596) in the ITT population (primary endpoint) and 0.34 (95.41% CI 0.11–1.00; *p* = 0.0345) in the PPS	
			In the open-label extension period, 46.4% of patients reduced the dose, which was less than one-half the dose administered at relapse before study entry.	
Wang *et al*.	JAK/STAT3	RCT	LEF and TOF were comparable for TAK treatment. TOF group had advantage of GC dose reduction while keeping a similar rate of persistent remission (46.88% vs. 17.14%)	[120]
2022	TOF	GC in addition to TOF vs. GC in addition to LEF		
Kong *et al*.	JAK/STAT3	RCT	TOF group had a greater advantage in CR rate (88.46% vs. 56.52%; *p* = 0.02), relapse rate (11.54% vs. 34.78%; *p* = 0.052), median relapse-free duration (11.65 ± 0.98 vs. 10.48 ± 2.31 months; *p* = 0.03), and average GC dose after 12 months of treatment.	[121]
2022	TOF	GC in addition to TOF vs. GC in addition to MTX		
			No statistical difference in side effects between the two groups.	
Wu *et al*.	TLR4-JAK2/AKT/STAT3	Clinical trial	Patients with Kerr score ≥1 decreased significantly during 3 months with the combination compared to baseline (75.0% vs. 31.2%; *p* = 0.03).	[58]
2021	Curcumin	Curcumin (7.5 g bid) in addition to GC, tacrolimus, LEF, AZA, MMF, Rapamycin and HCQ		
Régnier *et al*.	JAK/STAT3	Clinical trial	CD4+ effector T-cell activation or differentiation was reduced, expression by CD4+ T cells was reduced and Treg percentages were increased after 6 months of treatment compared with baseline.	[127]
2020	JAKi	GC and JAKi		
			CRP level at 6 months was reduced and treatment allowed GC dose reduction for two-thirds of patients and led to a reduction of NIH activity score to 0 for all treated patients compared with baseline.	

ADA, Adalimumab; AZA, Azathioprine; CI, Confidence interval; CR, Complete remission; CRP, C-reactive protein; ER, Efficacy rate; GC, Glucocorticoid; HCQ, Hydroxychloroquine; HR, Hazard ratio; IFX, Infliximab; IQR, Interquartile range; ITT, Intention-to-treat; JAKi, JAK inhibitor; LEF, Leflunomide; MMF, Mycophenolate mofetil; MTX, Methotrexate; NIH, National Institutes of Health; PPS, Per-protocol set; RCT, Randomized controlled trial; RR, Risk ratio; s.c., Subcutaneous; TCZ, Tocilizumab; TAK, Takayasu’s arteritis; TNFi, Tumor necrosis factor alpha inhibitor; TOF, Tofacitinib.

### 7.5 IL-1β Inhibitor

IL-1β is closely related to the pathogenesis of TAK and is involved in regulating the activation of IL-6. Canakinumab is a humanized monoclonal antibody and inhibitor targeting the IL-1 signaling pathway to exert anti-inflammatory effects and significantly reduces plasma levels of IL-6. The Canakinumab Anti-inflammatory Thrombosis Outcomes Study (CANTOS) demonstrated that administering 150 mg canakinumab every 3 months, a drug that targets the IL-1β pathway to reduce inflammation, significantly decreased the incidence of recurrent cardiovascular events compared to the placebo group. The mechanism by which canakinumab decreased the risk may involve downregulation of IL-1β associated with activation by cholesterol. Currently, canakinumab is being tested in clinical trials for atherosclerosis, diabetes, chronic kidney disease, gout, and some autoimmune diseases. Despite the lack of data on the use of canakinumab for TAK treatment, it has value as a potential anti-inflammatory therapeutic target.

In conclusion, TAK, is a rare but serious and complex inflammatory disease with a still largely unknown pathogenesis, posing a significant challenge to human health. While progress has been made in understanding the risk factors, pathogenesis mechanisms, pathological changes, and clinical features in TAK in recent years, the specific role of the NLRP3 inflammasome in the development of TAK needs further in-depth investigation to improve our knowledge and promote a novel targeted therapeutic strategy.

## 8. Conclusions

Recent studies have revealed the significant role of NLRP3 inflammasomes in various rheumatic diseases such as gouty arthritis. In comparison, TAK is rarer but has an equally profound impact, particularly on the younger population, with potential risks of disability and sudden death. In 2018, it was first confirmed that NLRP3 inflammasomes are expressed at elevated levels in cultured macrophages from patients with TAK. However, a limited number of follow-up studies and theoretical updates have been pursued since that time and numerous questions remain unanswered. First, HSP60 has been established as a priming signal for the NLRP3 inflammasome, and there is evidence suggesting that HSP65, due to its homology with HSP60, might also be involved in regulating the priming of NLRP3 inflammasomes, possibly through TLR4-related pathways. In addition, *in vitro* experiments have demonstrated that the *MLX* genotype diversity indirectly modulates the activation of NLRP3 inflammasomes by affecting oxidative stress mechanisms; however, there is a lack of animal model validation of this finding. *MLX* genotype does influence the process, but it remains unknown if *MLX* gene diversity plays a predominant role in the current prevalence of TAK. Furthermore, an interesting observation is that functional P2X7Rs can mediate the activation of NLRP3 inflammasomes, whereas non-functional receptors exhibit a higher expression frequency in patients with TAK combined with pulmonary tuberculosis. The P2X7R is involved in both the immune response to eliminating *M. tuberculosis* and plays a role in mediating the activation of NLRP3 inflammasomes. It remains unknown how the P2X7R and NLRP3 inflammasomes influence the occurrence of TAK combined with pulmonary tuberculosis. Existing theories still contain gaps that require in-depth investigations (Table 4). The study of these mechanisms will enhance the understanding of TAK and inflammation, potentially accelerating the discovery of novel therapeutic targets and interventions.

**Table 4.** **Role of NLRP3 inflammasomes in TAK: current status, unmet needs, and potential solutions**.

Current status	Unmet needs	Proposals and potential solution
Limited epidemiological data due to low incidence and prevalence rates of TAK	Analysis of the temporality between risk factors and disease onset	Implementation of a cohort study
High underdiagnosis rate of TAK due to nonspecific clinical presentation	Enhancement of clinician awareness of TAK	Screening of patients with similar clinical manifestations using angiography
Complexity of current induction methods and suboptimal fidelity in animal models	Animal models demonstrating superior hit rate, cost-effectiveness, and survival rate	Exploration and refinement of animal model establishment
Under investigated hereditary patterns of newly identified susceptibility genes	Lack of inheritance patterns and population distribution of emerging genetic variants including *SVEP1*, *CFL2*, *VPS8*, *chr13q21*, and *IL18-137G/C*	Pedigree investigation and genetic sequencing of probands
Unclear role of HSP65 in linking HSP60, *M. tuberculosis*, and TAK pathogenesis	Investigation of *M. tuberculosis* pathogenesis via the connection with HSP65 and HSP60 directly in TAK	Examining TLR4-mediated signaling pathway activation by stimulating *in vitro*-cultured neutrophils from TAK patients with HSP65
Limited *in vitro* evidence of the *MLX* gene in modulating NLRP3 inflammasome formation via oxidative stress in TAK	Delineation of the ROS-TXNIP-NLRP3 axis pathogenic role within TAK	Assessment of MLX-knockout animal model manifestations of the pathological features of TAK
Non-functional P2X7R may reduce NLRP3 inflammasome activation signals and confer immune privilege to *M. tuberculosis*, exhibiting a paradoxical dual relationship in TAK pathogenesis; however, direct experimental evidence remains lacking.	Research on the effect of non-functional P2X7R on NLRP3 inflammasome activation in a TAK model	Measure NLRP3 levels in neutrophils derived from patients with TAK following P2X7R blockade.
	Research on the relationship between non-functional P2X7R affecting *M. tuberculosis* clearance and TAK-related manifestation	Assess the pathogen clearance of TAK patient-derived P2X7R blocked macrophages after infecting with *M. tuberculosis*.

HSP65, Heat shock protein 65; P2X7R, P2X7 receptor; ROS, Reactive oxygen species; TAK, Takayasu’s arteritis; TLR4, Toll-like receptor 4; TXNIP, Thioredoxin-interacting protein.

## Abbreviations

ACPA, anti-citrullinated protein/peptide antibodies; APLs, altered peptide ligands; APCs, antigen-presenting cells; ASC, apoptosis-associated spot-like protein; CitH3, citrullinated histone H3; CRP, c-reactive protein; dsDNA, double-stranded DNA; ESR, erythrocyte sedimentation rate; eATP, extracellular ATP; eDNA, extracellular dsDNA; GSDMD, Gasdermin D; GC, glucocorticoid; HSP65, heat-shock protein 65; IL, interleukin; MMPs, matrix metalloproteinases; MTX, methotrexate; MPO, myeloperoxidase; NK cells, natural killer cells; NE, neutrophil elastase; NETs, neutrophil extracellular traps; NLRP3, NOD-like receptor pyrin domain-containing protein 3; PAMPs, pathogen-associated molecular patterns; PRR, pattern recognition receptor; PBMCs, peripheral blood mononuclear cells; PINK1, PTEN-induced kinase 1; P2X7R, P2X7 receptor; ROS, reactive oxygen species; RIPK1, receptor-interacting serine/threonine-protein kinase 1; RA, rheumatoid arthritis; TAK, Takayasu’s arteritis; TXNIP, thioredoxin-interacting protein; TLRs, Toll-like receptors; TNF, tumor necrosis factor.
